# Experimental Biology and medicine conference thematic issue introduction

**DOI:** 10.3389/ebm.2025.10698

**Published:** 2025-06-27

**Authors:** Warren Zimmer

**Affiliations:** Medical Physiology, College of Medicine, Texas A&M University Health Science Center, College Station, TX, United States

**Keywords:** cardiovascular, neuroscience, regenerative medicine, editorial, conference

The Society for Experimental Biology and Medicine (SEBM) sponsored a scientific meeting called Experimental Biology and Medicine Conference (EBMC 2024) on 13–16 October 2024. This was a transition for the Society from being a part of the larger Experimental Biology meeting to a meeting completely organized by SEBM and was the inaugural national meeting for the society. A committee of resolute members planned for over 2 years to have an in person meeting in Orlando, Florida. While planning touched on almost all contingencies, the track of Hurricane Milton through central Florida and Orlando necessitated a change of plans. In a tour de force SEBM changed the meeting format to online in just under 48 h to avoid any weather-related issues.

Even though EBMC moved to an online format, SEBM, a premier supporter of basic biomedical interdisciplinary research, maintained true to its mission of the dissemination of innovative translational research engaging basic and clinical scientists as well as promoting the career development of trainees and early career scientists. Over 50 scientists presented their cutting-edge research at EBMC and the Society, in collaboration with the Alliance for Cell Therapy, held a tribute to Dr. Arnold (Arnie) Caplan, the discoverer of Mesenchymal Stem Cells (MSCs) and largely regarded as the “father” of modern stem cell research therapies.

With input from SEBM membership, it was decided to have 3 themes for the meeting which were: Disorders of the Nervous System, Cardiovascular Disease and Regenerative Medicine: Stem Cell Based Therapies. Each theme was chosen to be an umbrella which sessions had content from basic science to clinical science. Each speaker was given the opportunity to submit a manuscript to the Journal (EMB) that summarized their work. The program committee chose the following papers to highlight in a special issue of the journal, termed *Experimental Biology and Medicine Conference Thematic Issue*, that represents at least one study from each area of interest from EBMC 2024.

Cardiovascular Diseases (CVDs) are a collection of disorders of the heart and blood vessels and remain a leading cause of death worldwide. One major issue is the inability of heart cells to be repaired after injury. This has led to many studies of stem cell therapies for heart repair, however there is limited positive outcomes from this approach. The studies described by [1] show that there is significant reprogramming of heart cells (cardiomyocytes) *in situ* through limited injection of synthetic mRNAs encoding two important developmental factors called STEMIN and YAPS5A. Short term expressions of these factors lead to a reprogramming and subsequent repairing of the once senescent cardiomyocyte. This approach represents a major leap forward in potential therapies of cardiac injuries such as myocardial infarction and heart failure.

A second principal CVD issue involves changes in vessels carrying blood from the heart to the periphery, largely referred to as Atherosclerosis. These changes are responsible for a range of cardiovascular and cerebrovascular diseases, such as heart attack, heart failure, and stroke and are a major contributor to the global burden of cardiovascular disease. The studies reported here by [2] describe the capability of reversing many of the debilitating changes of atherosclerotic disease through restoring copper homeostasis in the system. Thus, it is possible that new, improved therapies for reversing atherosclerosis can be had by simply repairing metal physiology to the affected cells and tissues.

Like cardiomyocytes, neural cells are particularly intransigent to regeneration and repair. This is especially important in treatment of issues such as spinal cord injury (SCI). As discussed in the review by [3] there have been huge discoveries in treating SCI. However, there remains barriers in developing therapies for such injuries. As discussed in their review there is great hope with the continued progress in the field aimed at enhancing quality of life and functional outcomes for patients with debilitating spinal cord injuries.

As mentioned above, EBM dedicated a session to a tribute for Dr. Arnie Caplan, the recognized founder of stem cell therapies. A theme of the Regenerative Medicine talks centered around the ability to generate stem cells sufficiently from patients to be used in attacking a specific disease or injurious issue. This is a major problem and leads to treating patients with non-autologous cells leading to important issues of rejection of the added cells by the hosts, sometimes leading to greater complications than the initial lesions. The studies reported here by [4] at the University of Tennessee Health Science Center shows that induced mesenchymal stem cells (MSCs) can be isolated from periodontal ligament tissues in numbers that allow for regenerative medicine treatments. Thus, these authors indicate that we may be on the threshold of designing therapies using cells from the patients themselves. Clearly a breakthrough in the ability of treating many diseases and improving world health.

The papers presented here in the *Experimental Biology and Medicine Conference Thematic Issue* represent a small sample of the cutting-edge research presented at EBM 2024. Although we did switch to a virtual format, the presentations provoked much discussion and have led to enhanced collaborations among scientists that might not have come together except for listening and participating in the diverse talks.

Respectfully,

Warren Zimmer, PhD, SEBM Past President
